# Skill Enactment Among University Students Using a Brief Video-Based Mental Health Intervention: Mixed Methods Study Within a Randomized Controlled Trial

**DOI:** 10.2196/53794

**Published:** 2024-08-21

**Authors:** Hayley M Jackson, Philip J Batterham, Alison L Calear, Jeneva L Ohan, Louise M Farrer

**Affiliations:** 1 Centre for Mental Health Research National Centre for Epidemiology and Population Health The Australian National University Acton ACT Australia; 2 School of Psychological Science University of Western Australia Perth Australia; 3 Telethon Kids Institute Nedlands Australia

**Keywords:** university students, young people, internet, computer-assisted therapy, engagement, skill enactment, depression, anxiety, randomized controlled trial, mobile phone

## Abstract

**Background:**

Mental health problems are common among university students, yet many students do not seek professional help. Digital mental health interventions can increase students’ access to support and have been shown to be effective in preventing and treating mental health problems. However, little is known about the extent to which students implement therapeutic skills from these programs in everyday life (ie, skill enactment) or about the impact of skill enactment on outcomes.

**Objective:**

This study aims to assess the effects of a low-intensity video-based intervention, Uni Virtual Clinic Lite (UVC-Lite), in improving skill enactment relative to an attention-control program (primary aim) and examine whether skill enactment influences symptoms of depression and anxiety (secondary aim). The study also qualitatively explored participants’ experiences of, and motivations for, engaging with the therapeutic techniques.

**Methods:**

We analyzed data from a randomized controlled trial testing the effectiveness of UVC-Lite for symptoms of depression and anxiety among university students with mild to moderate levels of psychological distress. Participants were recruited from universities across Australia and randomly assigned to 6 weeks of self-guided use of UVC-Lite (243/487, 49.9%) or an attention-control program (244/487, 50.1%). Quantitative data on skill enactment, depression, and anxiety were collected through baseline, postintervention, and 3- and 6-month follow-up surveys. Qualitative data were obtained from 29 intervention-group participants through open*-*ended questions during postintervention surveys (n=17, 59%) and semistructured interviews (n=12, 41%) after the intervention period concluded.

**Results:**

Mixed model repeated measures ANOVA demonstrated that the intervention did not significantly improve skill enactment (*F*_3,215.36_=0.50; *P*=.68). Skill enactment was also not found to influence change in symptoms of depression (*F*_3,241.10_=1.69; *P*=.17) or anxiety (*F*_3,233.71_=1.11; *P*=.35). However, higher levels of skill enactment were associated with lower symptom levels among both intervention and control group participants across time points (depression: *F*_1,541.87_=134.61; *P*<.001; anxiety: *F*_1,535.11_=73.08; *P*<.001). Inductive content analysis confirmed low levels of skill enactment among intervention group participants. Participants were motivated to use techniques and skills that were perceived to be personally relevant, easily integrated into daily life, and that were novel or had worked for them in the past.

**Conclusions:**

The intervention did not improve skill enactment or mental health among students with mild to moderate psychological distress. Low adherence impacted our ability to draw robust conclusions regarding the intervention’s impact on outcomes. Factors influencing skill enactment differed across individuals, suggesting that it may be necessary to tailor therapeutic skills and engagement strategies to the individual user. Theoretically informed research involving collaboration with end users is needed to understand the processes underlying skill enactment in digital mental health interventions.

**Trial Registration:**

Australian New Zealand Clinical Trials Registry ACTRN12621000375853; https://tinyurl.com/7b9ar54r

## Introduction

### Background

University students are a population in need of mental health support. Before the COVID-19 pandemic, findings from the World Health Organization World Mental Health International College Student initiative indicated that approximately one-third of students met the criteria for a mental disorder each year [1,2]. Mood and anxiety disorders are the most common diagnoses [1]. Research conducted since the beginning of the pandemic has generally indicated an increase in the prevalence of mental health problems among university students [3,4]. It is estimated that 30% to 40% of students experienced elevated levels of depression and anxiety symptoms during the first 2 years of the pandemic alone [5,6], with the highest rates reported for students in low- and middle-income countries [4]. Mental health problems among students have been associated with a range of negative outcomes, including reduced academic performance, lower engagement in campus life, poorer interpersonal relationships, and higher dropout rates [2,7-9]. However, evidence from upper-middle and high-income countries suggests that only 25% of students with a mental disorder receive treatment in a given year [2]. Common barriers to accessing mental health services include lack of time, stigma, high treatment costs, and insufficient capacity of university counseling centers to meet student demand [10,11]. Some of these barriers may be addressed through the provision of evidence-based digital mental health interventions (DMHIs) [2,12].

University students from various countries and regions have indicated a willingness to use digital tools for mental health–related information and support, highlighting benefits in improved access, privacy, confidentiality, and reduced costs [13]. Moreover, several systematic reviews have indicated that DMHIs targeting tertiary students can be effective for symptoms of depression and anxiety when delivered under trial conditions [12,14,15], with the most robust support for programs based on cognitive behavioral therapy (CBT) [12]. However, there is also evidence to suggest that the benefits of DMHIs are diminished in real-world settings [16,17], with high rates of dropout and a lack of sustained engagement frequently reported as key challenges to program effectiveness and successful implementation in both the general and student populations [15,18]. Although strategies such as the use of reminders, coaching, and engagement facilitation interventions offer the potential to redress suboptimal levels of engagement [19-21], their effectiveness has so far been variable, and a recent systematic review concluded that more research is needed to ensure that engagement strategies are effective and acceptable for end users [22].

Efforts to improve engagement with DMHIs are further complicated by a lack of consensus regarding how to define and measure engagement [22]. Most previous studies have focused on program use indicators of engagement (eg, number of logins or modules accessed) [23,24], reflecting the extent to which users are exposed to or interact with intervention materials. These indicators provide relatively objective information on use patterns and have been associated with small to moderate improvements in mental health outcomes in some instances [25,26]. However, they may not account for other factors that might be more closely related to positive outcomes, such as the degree to which users integrate and apply the therapeutic techniques from an intervention into their daily lives (ie, skill enactment) [27]. Recent recommendations, such as those provided by Li et al [24], Beintner et al [23], and Baumel [27], underscore the importance of investigating skill enactment as a distinct aspect of engagement that goes beyond the technology used to capture the cognitive and behavioral changes a user makes in their everyday life because of intervention [27-30]. Evaluating the strategies and techniques individuals implement from digital interventions is likely to be important both as a stand-alone outcome following intervention and because of its potential role as a mediator of treatment outcomes [31,32]. Skill enactment is likely to be an important outcome to consider in itself given suggestions that adopting healthy actions and routines may help maintain mental health or prevent future problems by improving the individual’s ability to cope with stressors [31,32]. This may be especially relevant in interventions targeting relatively mild distress or symptoms where improvements in mental health outcomes may be small. Moreover, skill enactment has also been argued to be critical to understanding the impact of an intervention on symptom reduction [27,33]. This is because most therapeutic programs are predicated on the theoretical understanding that positive change occurs by facilitating the uptake of adaptive cognitive and behavioral skills [27].

The proposed importance of skill enactment has some support from recent studies that have demonstrated that DMHIs can effectively increase levels of skill enactment among adults with at least clinically mild symptoms of depression or anxiety at mid or postintervention assessments [34-36]. These studies also tend to indicate that improvements in skill enactment (ie, increase in the use of cognitive or behavioral skills, such as cognitive reframing and behavioral activation) are associated with small to moderate improvements in depression and anxiety [35-38]. However, a recent systematic review of digital CBT programs targeting depression and anxiety identified a gap in the literature on skill enactment among young people [30], and none of the included studies explored changes in skill enactment among a university student sample. Furthermore, qualitative or mixed methods investigations of skill enactment in digital interventions are rare [39], and we are not aware of any studies specifically exploring experiences of skill enactment among university students. As little is known about skill enactment in this population, there is a need for research examining skill enactment, its potential implications for mental health outcomes, and experiences among university students participating in DMHIs.

### This Study

This mixed methods study investigated skill enactment among university students with mild to moderate distress who participated in a randomized controlled trial (RCT) of the Uni Virtual Clinic Lite (UVC-Lite), a low-intensity internet-based program designed to address depression, anxiety, and other mental health problems that commonly affect university students. The results from the trial have been submitted for publication [40] and indicated no differences between the UVC-Lite intervention and an attention-control program on any of the primary (symptoms of depression and generalized anxiety disorder) or secondary outcomes at postintervention measurement, 3-month, or 6-month follow-ups. Despite these nonsignificant results, understanding patterns and experiences related to skill enactment remains pertinent due to its potential relevance in maintaining mental health and improving future interventions. The primary aim of this study was to investigate whether the UVC-Lite intervention led to improvements in skill enactment. As per the study protocol, we also intended to investigate whether improvements in skill enactment mediated change in mental health outcomes (secondary aim). Although the intervention was not shown to be effective, we retained statistical analyses to explore the association between skill enactment and mental health outcomes. As there was a significant reduction in symptoms of depression and anxiety at posttest assessment across both conditions, there remained the potential to observe whether change in mental health outcomes was associated with skill enactment. We restricted our analysis to investigate the primary outcomes of depression and anxiety symptoms. We supplemented the quantitative component with partial data set analysis of semistructured interviews and free-text response data from participants who received access to UVC-Lite to examine patterns of skill enactment and explore participants’ motivations for using the techniques delivered in the program. By collecting feedback from participants, it may be possible to refine the content and features of the intervention to enhance engagement and skill enactment.

We hypothesized that skill enactment would be higher in the UVC-Lite intervention condition than the attention-control condition at postintervention, 3-month, and 6-month follow-up assessments.

## Methods

### Overview

The CONSORT-EHEALTH (Consolidated Standards of Reporting Trials of Electronic and Mobile Health Applications and Online Telehealth) is presented in Multimedia Appendix 1 [41]. Data for this study were collected as part of an RCT that examined the effectiveness of UVC-Lite in reducing depression and anxiety symptoms among university students with mild to moderate levels of psychological distress. Participants were randomly assigned to either the UVC-Lite intervention or an attention-control program for a period of 6 weeks. A full description of the trial methods and primary outcomes, including information on allocation concealment, randomization procedures, and blinding, is provided in the main trial paper [40].

### Participants

#### RCT Data

Trial participants were 487 undergraduate and postgraduate students, recruited and randomized into the trial between August 2021, and May 2022. Follow-up data were collected by January 2023. Students from all universities in Australia were invited to participate in the trial using a range of recruitment methods. These included targeted social media advertising, posts in university-related social media groups, and advertisements in university newsletters. In addition, assistance was sought from student associations and advocacy departments, student housing services, university marketing teams, survey management departments, university counseling and well-being clinics, and academic course coordinators to disseminate information about the trial to students via email and flyers. A total of 2865 students clicked on the study invitation and were screened for eligibility. Of these, 523 (18.25%) participants were initially screened into the trial, completed baseline measures, and were randomized to the UVC-Lite intervention (n=261, 49.9%) or attention-control group (n=262, 50.1%). A further 6.9% (36/523) of the participants were excluded after randomization due to being older than the eligible age range. Respondents were eligible for inclusion if they were (1) enrolled at an Australian university and resided in Australia, (2) aged between 18 and 25 years, and (3) scored between 8 and 17 on the Distress Questionnaire-5, indicating mild to moderate levels of psychological distress [42]. Students diagnosed with bipolar disorder, schizophrenia, posttraumatic stress disorder, or a personality disorder could participate if they met eligibility criteria and were receiving support or treatment for their disorder. Students who were ineligible due to high levels of psychological distress were provided with help-seeking resources.

#### Follow-Up Interviews

Purposive sampling was used to recruit participants for interview. All participants (131/243, 53.9%) randomized to the intervention condition who completed the postintervention survey were invited to provide additional feedback by telephone or videoconference about their experience with the modules. Of these, 38.9% (51/131) of the respondents indicated a willingness to provide their feedback. From this group, 33% (17/51) of the participants responded to the subsequent email invitation and 24% (12/51) participated in an interview. Reasons for not participating included loss of contact (3/51, 6%) and being overseas (2/51, 4%). In addition, 1 follow-up email was sent to participants if there was no response to the initial email. No incentives were offered for participation in the interview.

### Intervention and Attention-Control Conditions

#### Intervention Condition: UVC-Lite

The UVC-Lite is a low-intensity internet-based intervention comprising 12 modules that target the common mechanisms underlying mental health problems in university students. Content for the intervention was drawn from the Uni Virtual Clinic, an web-based mental health intervention for university students that was shown to be effective in reducing symptoms of social anxiety and improving academic self-efficacy compared to a waitlist control group in a previous trial [43,44]. Each UVC-Lite module comprises a 3- to 6-minute video presenting psychoeducation about a mental health problem and a therapeutic technique designed to address the problem. The videos provide practical examples and guidance on how to apply the techniques in daily life. The modules also include optional exercises designed to encourage the practice of therapeutic techniques, self-monitoring quizzes (included in modules 1, 3, 4, 5, 7, and 11), and links to help-seeking resources provided at their university or in the community if necessary. The twelve modules include (1) dealing with depression and low mood (behavioral activation), (2) tackling negative and anxious thoughts (cognitive reframing), (3) dealing with anxiety (cognitive reframing, breathing, and grounding techniques), (4) managing study issues: procrastination and time management (practical strategies for time management), (5) perfectionism (challenging perfectionistic thoughts and behaviors), (6) coping with stress (mindfulness practice), (7) managing sleep issues (sleep hygiene), (8) social anxiety and shyness (behavioral experiments and exposure techniques), (9) relationships and loneliness (communication skills and social support), (10) social media use (practical strategies for reducing social media use), (11) body image (body functionality appreciation), and (12) thoughts of suicide (safety planning). Modules were delivered twice weekly over the 6-week intervention period.

#### Attention-Control Condition: General Health Information

Participants assigned to the attention-control condition received 2 emails per week containing a web link to a PDF document providing information on topics related to general health rather than mental health, approximately matched for completion time with the active intervention. A total of 12 topics were covered, including bone health, sun exposure, food hygiene, dietary supplements, kidney health, microbes, household burns, respiratory viruses, heart health, allergens, posture, and pancreas health. A similar program has previously been shown to be associated with no therapeutic reductions in depression [45].

### Data Collection

#### Overview

At baseline, the following demographic data were collected: age, gender, ethnicity, living situation, employment status, study discipline and year of degree, study load, and international or domestic student status. Quantitative data on skill enactment, depression symptoms, and generalized anxiety symptoms were obtained using web-based self-report questionnaires at baseline, postintervention assessment (emailed to participants 6 weeks after completion of the baseline survey), and 3- and 6-month follow-ups (emailed to participants 3 and 6 months after receiving the postintervention survey). Participants received 2 email reminders to finish the postintervention and follow-up surveys. Qualitative data were collected at postintervention assessments (free-text responses) and through interviews conducted with participants following the completion of postintervention assessments between April and August 2022.

#### Skill Enactment

We assessed skill enactment using a list of 14 items developed to assess the frequency of enacting therapeutic techniques from the UVC-Lite program (eg, “I made time for activities that make me feel better” and “I challenged my thinking to be more realistic and helpful”). At each time point, participants rated how often they used each of the skills over the past 2 weeks on a 5-point scale using the following response options: 0 (never), 1 (rarely), 2 (sometimes), 3 (often), and 4 (very often). Item development was informed by existing CBT skills use questionnaires [46,47]. Exploratory factor analysis was conducted on the skill enactment items before computing total scores. The results of this analysis indicated that the scale could be treated as a single factor. One item was dropped from the measure due to a low factor loading, resulting in a 13-item measure (Multimedia Appendix 2 [48-50]). The internal consistency of the scale was acceptable (Cronbach α=0.84). The 13 items yielded a summed total score ranging from 0 to 52. Higher scores indicate more frequent skill enactment.

#### Mental Health Outcomes

Primary mental health outcomes were symptoms of depression, measured by the Patient Health Questionnaire-9 (PHQ-9) [51], and generalized anxiety disorder, measured by the Generalized Anxiety Disorder-7 (GAD-7) scale [52]. The PHQ-9 comprises 9 items rated on a 4-point scale, ranging from 0 (not at all) to 3 (nearly every day). Item scores are summed to produce an overall severity score ranging from 0 to 27, with higher scores indicating greater symptom severity (0-4: no symptoms, 5-9: mild symptoms, 10-14: moderate symptoms, and 15-27: severe symptoms). The GAD-7 comprises 7 items rated on the same 4-point scale as the PHQ-9. Summed scores produce an overall severity score ranging from 0 to 21 (0-4: no symptoms, 5-9: mild symptoms, 10-14: moderate symptoms, and 15-21: severe symptoms). Higher scores indicate greater symptom severity. Both scales have demonstrated robust psychometric properties in general population and university student samples [53-56]. In the current sample, internal consistency was acceptable at baseline (PHQ-9: Cronbach α=0.80; GAD-7: Cronbach α=0.84).

#### Program Use

JavaScript code was used to track intervention group participants’ uptake and use behaviors. We report specific indicators in this study to provide an indication of exposure to the therapeutic content, including the number of modules accessed (ie, whether participants clicked to open each module), number of videos started (ie, >0% of the video watched), and number of therapeutic activities accessed (based on whether participants clicked through to access the exercise). For the control group, we tracked the number of modules accessed.

#### Qualitative Data

Semistructured interviews were conducted by the first author (HMJ), with ongoing supervision provided by the senior author (LMF). Overall, 7 interviews were conducted via Zoom (Zoom Video Communications), a web-based teleconferencing platform, and the remaining 5 interviews were conducted via telephone. The interview guide was developed by the researchers to investigate participants’ thoughts about and use of the video modules and exercises, their suggestions for implementation in universities, and any barriers to the modules being used by university students. The full list of interview questions is provided in Multimedia Appendix 3, although only a subset of questions was the focus of investigation in this paper. Interviews were audio-recorded and transcribed verbatim using a speech-to-text transcription application (Otter.ai; Otter.ai, Inc). Transcripts were corrected by the first author while listening to the audio-recorded interviews.

Participants’ satisfaction with and views of the intervention were also assessed during postintervention surveys by asking participants open-ended questions regarding whether there was anything they liked about the program, whether there was anything they disliked about the program, and whether they would change anything about the program.

### Data Analysis

#### Quantitative Data

Quantitative data were analyzed using SPSS for Windows (version 28.0; IBM Corp), and significance was set at *P*<.05. Primary analyses were undertaken on an intention-to-treat basis. Mixed model repeated measures (MMRM) ANOVA was conducted to compare mean scores on skill enactment between the intervention and control groups, with measurement occasion as a within-groups factor and condition as a between-groups factor. MMRM techniques incorporate all available data under the assumption that data are missing at random [57]. Within-person variation was modeled using unstructured covariance matrices, and *df* was estimated using Satterthwaite correction. Subgroup analyses of intervention group participants who started ≥1 videos versus all of the control group were conducted to estimate the effectiveness of UVC-Lite among participants who accessed at least some of the therapeutic content. We opted to use this indicator of program use in the analyses as use of the videos was considered sufficient to promote acquisition and enactment of the therapeutic skills in this intervention and because the module exercises were optional. The influence of skill enactment on primary outcomes (PHQ-9 and GAD-7) was explored using MMRM ANOVA, including 3-way interaction terms for time×condition×skill enactment, as well as all main effects and 2-way interactions. Time-varying skill enactment, measured simultaneously with mental health outcomes, was used in the models. The 3-way interaction tested whether changes in outcome over time differed more for those in the intervention group with greater skill enactment, relative to the control group. The time × skill enactment interaction tested how a change in skill enactment was related to symptoms across different time points.

#### Qualitative Data

Qualitative data were analyzed using inductive content analysis [58,59], as per the analytic steps outlined by Vears and Gillam [60]. This approach is well suited to answering applied research questions [60]. After reading each of the interviews and free-text responses, the first author extracted all data potentially relevant to the research question to develop a working data set (ie, extracts referring to any aspects of in-program exercises or skill enactment) [61]. Data extracts were drawn from all (12/12, 100%) interview participants and 16.5% (17/103) of the participants who provided free-text responses during the postintervention survey, resulting in the analysis of data from 11.9% (29/243) of intervention group participants. The data then went through several iterations of inductive coding and similar codes were clustered together into content categories. Reporting was structured around the categories, drawing on illustrative quotes from interview transcripts and open-ended responses. Data extracts are presented verbatim, though some words (eg, “like” and “um”) were removed to improve readability. Participants are identified by their gender, a coded number, and the type of data provided (eg, M1, interview=male, interview participant 1). Quirkos (offline version; Quirkos Software) was used to analyze qualitative data.

### Ethical Considerations

The trial was registered at the Australian New Zealand Clinical Trials Registry (ACTRN12621000375853) and the ethical aspects of the research were approved by the Australian National University Human Research Ethics Committee (protocol #2020/412). All participants provided informed consent by ticking a checkbox on the web after viewing an information sheet containing key details about the study. To protect participant privacy, all data were deidentified. Participants who completed all 4 surveys were entered into a prize draw to win 1 of 10 Aus $100 (US $66.7) gift vouchers.

## Results

### Sample Characteristics and Participation Rates

**Figure 1:**
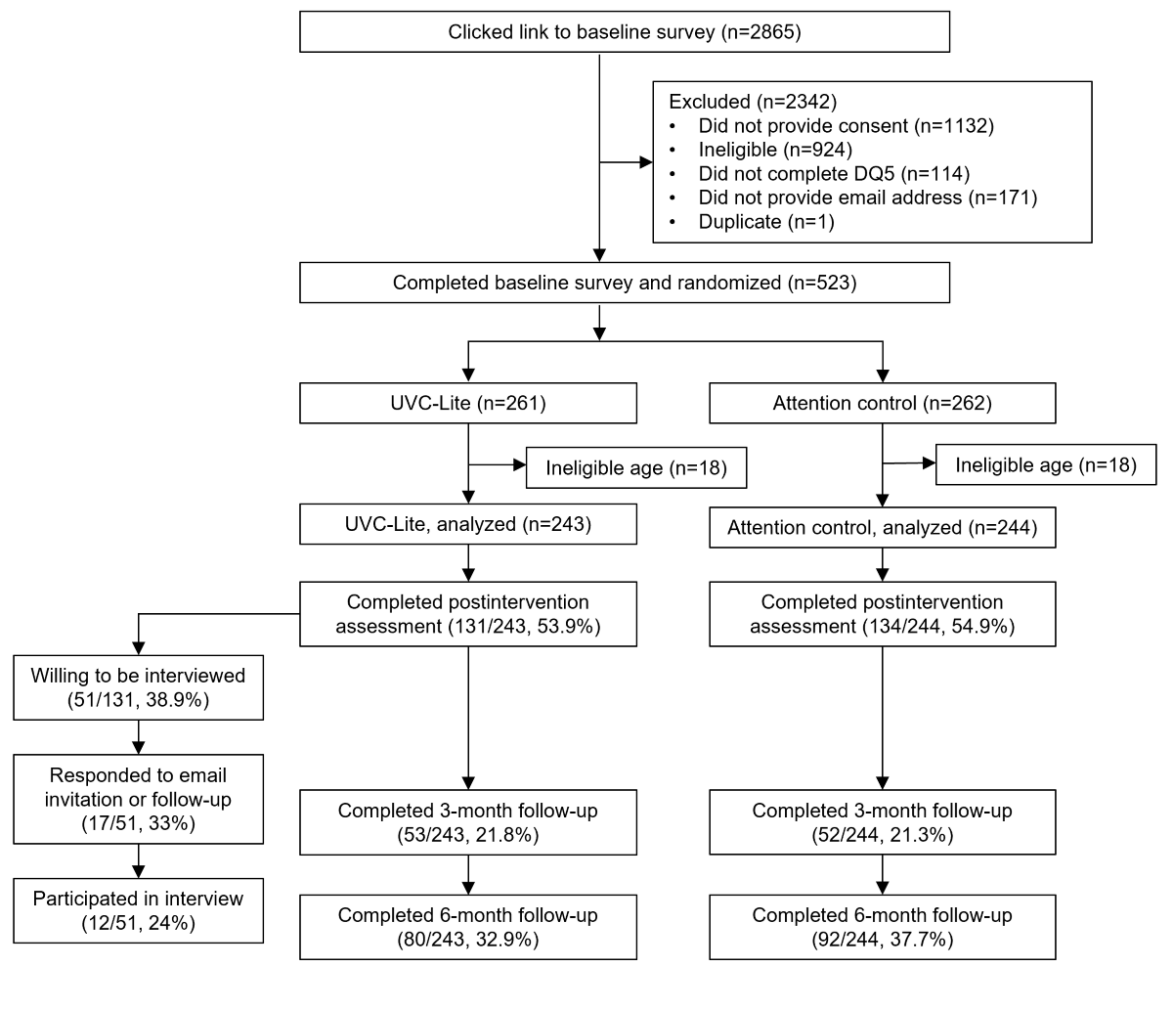
Flow of participants through the study. This figure is an abbreviated version of the flow diagram presented in the main trial paper by Farrer et al [40], which is published under Creative Commons Attribution 4.0 International License [62]. The figure has been adapted to include numbers on interview participation. DQ5: Distress Questionnaire-5; UVC-Lite: Uni Virtual Clinic Lite.

The flow of participants through the study is presented in Figure 1 [40,62]. Of the 487 students who were eligible, randomized, and completed baseline measures, 265 (54.4%) completed postintervention surveys, 105 (21.6%) completed 3-month follow-up surveys, and 172 (35.3%) completed 6-month follow-up surveys. Table 1 presents the demographic data for the full sample by trial condition. Students from 40 academic institutions across Australia participated. Most participants were female (355/487, 72.9%), with an average age of 20.62 (SD 2.12) years. Participants were predominantly domestic students (411/487, 84.4%) living either with family (209/487, 42.9%) or in on-campus accommodation (152/487, 31.2%). Most (328/487, 67.4%) participants were in some form of paid employment. Most students were studying full time (449/487, 92.2%) for an undergraduate degree (416/487, 85.4%); *t* tests (2-tailed) and chi square analyses demonstrated that there were no significant differences between the intervention and control conditions on any of the baseline demographic variables. Similarly, we found no significant differences between conditions on each of the outcomes of interest at baseline: skill enactment (t_483_=−1.06; *P*=.29), depression (t_471.98_=1.59; *P*=.11), and anxiety (t_485_=1.41; *P*=.16). At preintervention, skill enactment was negatively correlated with both depression (*r*=−0.35; *P*<.001) and generalized anxiety (*r*=−0.21; *P*<.001) symptoms.

**Table 1:** Sample characteristics by condition.

Characteristic		UVC-Lite^a^ (n=243)	Attention control (n=244)	Chi-square (*df*)		*t* test (*df*)		*P* value	
**Gender, n (%)**					0.4 (2)		—^b^		.82
	Male	52 (21.4)	58 (23.8)						
	Female	180 (74.1)	175 (71.7)						
	Other	11 (4.5)	11 (4.5)						
**Ethnicity, n (%)**					3.2 (4)		—		.53
	Aboriginal and/or Torres Strait Islander, or Pacific Islander	6 (2.5)	2 (0.8)						
	African or Middle Eastern	6 (2.5)	6 (2.5)						
	Asian or Indian	65 (26.7)	75 (30.7)						
	Caucasian or European	154 (63.4)	152 (62.3)						
	Other	11 (4.5)	8 (3.3)						
**Current living situation, n (%)**					4.6 (6)		—		.59
	With parents or family	100 (41.2)	109 (44.7)						
	On-campus housing	82 (33.7)	70 (28.7)						
	Friends off-campus	24 (9.9)	21 (8.6)						
	Others off-campus	19 (7.8)	18 (7.4)						
	With partner, children, or both	10 (4.1)	17 (7)						
	Alone	7 (2.9)	9 (3.7)						
	Other	1 (0.4)	0 (0)						
**Hours per week in paid employment, n (%)**					6.1 (5)		—		.30
	None	89 (36.6)	70 (28.7)						
	1-9	53 (21.8)	58 (23.8)						
	10-19	68 (28)	67 (27.5)						
	20-29	24 (9.9)	33 (13.5)						
	30-39	8 (3.3)	15 (6.1)						
	≥40	1 (0.4)	1 (0.4)						
**Discipline of degree studied, n (%)^c^**					5.5 (7)		—		.60
	Health/medicine	83 (38.4)	71 (32)						
	Arts/social sciences	37 (17.1)	42 (18.9)						
	Science	39 (18.1)	45 (20.3)						
	Engineering/computing	21 (9.7)	28 (12.6)						
	Business/economics	11 (5.1)	14 (6.3)						
	Law/criminology	14 (6.5)	15 (6.8)						
	Education	11 (5.1)	6 (2.7)						
	Tertiary preparation course	0 (0)	1 (0.5)						
**Year of degree, n (%)^d^**					4.3 (3)		—		.23
	First-year undergraduate	93 (38.4)	86 (35.4)						
	Later-year undergraduate	107 (44.2)	112 (46.1)						
	Honors	5 (2.1)	13 (5.3)						
	Postgraduate	37 (15.3)	32 (13.2)						
**Study load, n (%)**					0.4		—		.51
	Full time	226 (93)	223 (91.4)						
	Part time	17 (7)	21 (8.6)						
**Student status, n (%)**					0.1		—		.79
	Domestic	204 (84)	207 (84.8)						
	International	39 (16)	37 (15.2)						
Age (y), mean (SD)		20.67 (2.15)	20.57 (2.09)	—		−0.55 (485)		.58	

^a^UVC-Lite: Uni Virtual Clinic Lite.

^b^Not applicable.

^c^Discipline of degree data provided by 216 participants in the UVC-Lite condition and 222 participations in the attention-control condition.

^d^Year of degree data provided by 242 participants in the UVC-Lite condition and 243 participants in the attention-control condition.

### Program Use

The use of the UVC-Lite intervention among intervention group participants was low. Although 69.1% (168/243) of the participants accessed ≥1 (mean 5.10, SD 5.08) modules, more than half (129/243, 53.1%; mean 2.48, SD 3.89) of the participants did not start any of the videos, 28.8% (70/243) started 1 to 5 videos, 12.3% (30/243) started 6 to 11 videos, and only 5.8% (14/243) started all 12 videos. Engagement with the therapeutic activities was lower than engagement with the videos. Most participants (157/243, 64.6%; mean 1.31, SD 2.66) did not access any of the therapeutic activities; 27.2% (66/243) accessed 1 to 5 activities, and only 8.2% (20/243) of participants accessed ≥6 activities. Participants who received access to the attention-control program accessed a mean of 4.78 (SD 5.05) modules. More information on patterns of program use is provided in the study by Farrer et al [40].

### Change in Skill Enactment

Table 2 presents the observed means and SDs for the outcomes of interest at each measurement occasion across the 2 conditions, and Table 3 provides the estimates of fixed effects from the MMRM ANOVA models. There was no significant overall interaction effect between measurement occasion and condition for skill enactment (*F*_3,215.36_=0.50; *P*=.68) based on MMRM analyses. Planned contrasts demonstrated no significant interactions between time and condition on skill enactment scores at postintervention, 3-month, or 6-month follow-up assessments, indicating no differences in skill enactment between conditions. When subgroup analyses among those who started ≥1 videos (vs. the control group) were conducted, the overall effect of the intervention on skill enactment scores remained nonsignificant (*F*_3,179.78_=2.08; *P*=.11). However, exploratory post hoc contrasts demonstrated that intervention group participants who accessed ≥1 videos had higher levels of skill enactment than participants in the control condition at postintervention (t_231.96_=1.99; *P*=.048; 95% CI 0.02-4.01) and 6-month follow-up assessment (t_186.84_=2.09; *P*=.04; 95% CI 0.13-4.64), though not at 3-month follow-up (t_132.85_=0.58; *P*=.57; 95% CI –2.07 to 3.77). We also explored whether a change in skill enactment over time was modified by reliable improvement (reliable improvement vs no reliable improvement. Reliable improvement was defined as a decrease of ≥4 points on either the PHQ-9 or GAD-7 scales from pre to postintervention). The interaction between time, condition, and improvement was not significant (*F*_3,207.14_=1.13; *P*=.34), but there was a significant 2-way interaction between time and improvement (*F*_3,207.14_=8.78; *P*<.001). Post hoc contrasts indicated that, regardless of condition, participants demonstrating reliable improvement reported greater skill enactment at postintervention assessment (t_282.72_=3.97; *P*<.001; 95% CI 2.53-7.52) and 3 months (t_133.09_=2.02; *P*=.045; 95% CI 0.09-8.23) than those who did not. This was not significant at the 6-month follow-up (t_216.90_=1.81; *P*=.07; 95% CI –0.23 to 5.39).

**Table 2:** Observed means for skill enactment, depression, and anxiety scores at baseline, postintervention, and at 3- and 6-month follow-up.

Outcome			UVC-Lite^a^				Attention control		
			Participants, n (%)		Scores, mean (SD)		Participants, n (%)		Scores, mean (SD)
**Skill enactment**									
	Baseline	243 (100)		27.25 (8.24)		242 (99.2)		26.46 (8.09)	
	Postintervention	124 (51.0)		27.76 (7.31)		129 (52.9)		26.57 (8.14)	
	3-month follow-up	52 (21.4)		26.52 (8.97)		50 (20.5)		27.00 (9.14)	
	6-month follow-up	78 (32.1)		27.47 (7.12)		91 (37.3)		26.27 (6.54)	
**Depression (PHQ-9^b^)**									
	Baseline	243 (100)		9.70 (5.11)		244 (100)		9.02 (4.34)	
	Postintervention	131 (53.9)		9.04 (5.31)		134 (54.9)		8.24 (4.80)	
	3-month follow-up	53 (21.8)		9.06 (5.92)		52 (21.3)		8.13 (4.57)	
	6-month follow-up	80 (32.9)		9.58 (6.10)		92 (37.7)		9.02 (5.60)	
**Generalized anxiety (GAD-7^c^)**									
	Baseline	243 (100)		7.84 (4.53)		244 (100)		7.28 (4.13)	
	Postintervention	130 (53.5)		6.75 (4.19)		134 (54.9)		6.03 (3.90)	
	3-month follow-up	53 (21.8)		7.21 (4.84)		52 (21.3)		6.60 (4.36)	
	6-month follow-up	80 (32.9)		8.15 (5.52)		92 (37.7)		7.20 (4.77)	

^a^UVC-Lite: Uni Virtual Clinic Lite.

^b^PHQ-9: Patient Health Questionnaire-9.

^c^GAD-7: Generalized Anxiety Disorder Scale-7.

**Table 3:** Estimates of fixed effects from mixed model repeated measures models.

Outcome and source		*F* test (*df*)	*P* value
**Skill enactment**			
	Intercept	5607.77 (1, 357.91)	<.001
	Time	0.72 (3, 215.36)	.54
	Condition	1.10 (1, 357.91)	.29
	Time×condition	0.50 (3, 215.36)	.68
**Depression score (PHQ-9^a^)**			
	Intercept	644.17 (1, 567.48)	<.001
	Time	0.36 (3, 229.22)	.78
	Condition	3.30 (1, 567.48)	.07
	Skill enactment	134.61 (1, 541.87)	<.001
	Time×condition	1.68 (3, 229.22)	.17
	Time×skill enactment	1.19 (3, 241.10)	.31
	Condition×skill enactment	1.36 (1, 541.87)	.24
	Time×condition×skill enactment	1.69 (3, 241.10)	.17
**Generalized anxiety score (GAD-7^b^)**			
	Intercept	419.26 (1, 544.66)	<.001
	Time	2.05 (3, 221.68)	.11
	Condition	0.23 (1, 544.66)	.63
	Skill enactment	73.08 (1, 535.11)	<.001
	Time×condition	1.13 (3, 221.68)	.34
	Time×skill enactment	1.16 (3, 233.71)	.32
	Condition×skill enactment	0.15 (1, 535.11)	.70
	Time×condition×skill enactment	1.11 (3, 233.71)	.35

^a^PHQ-9: Patient Health Questionnaire-9.

^b^GAD-7: Generalized Anxiety Disorder Scale-7.

### Influence of Skill Enactment on Primary Outcomes

In MMRM models testing the effect of skill enactment on mental health outcomes, the 3-way interaction between time, condition, and skill enactment was not significant (PHQ-9: *F*_3,241.10_=1.69; *P*=.17; GAD-7: *F*_3,233.71_=1.11; *P*=.35), indicating no differential effects of the intervention for those with greater skill enactment. The 2-way interactions between time and skill enactment were also nonsignificant (PHQ-9: *F*_3,241.10_=1.19; *P*=.32; GAD-7: *F*_3,233.71_=1.16; *P*=.32). Planned contrasts confirmed these null results. However, a significant main effect of skill enactment was demonstrated on both depression (*F*_1,541.87_=134.61; *P*<.001) and anxiety (*F*_1,535.11_=73.08; *P*<.001) scores, indicating that higher levels of skill enactment were associated with lower symptom levels among both intervention and control group participants across time points. We also examined the influence of skill enactment among intervention group participants who started ≥1 videos (compared against all of the control group). While the main effect of skill enactment remained significant (PHQ-9: *F*_1,492.97_=130.47; *P*<.001; GAD-7: *F*_1,501.66_=071.95; *P*<.001), the 3-way interaction (PHQ-9: *F*_3,211.95_=1.47; *P*=.23; GAD-7: *F*_3,204.92_=0.9; *P*=.44) and the 2-way interaction effects between time and skill enactment (PHQ-9: *F*_3,211.95_=1.28; *P*=.28; GAD-7: *F*_3,204.92_=0.9; *P*=.44) was not significant.

### Qualitative Analysis

#### Description of Participants

Students from 18 academic institutions were represented in the qualitative analysis, 79% (23/29) of whom were female. The mean age of participants who provided qualitative data was 20.97 (SD 2.23) years. Most participants were domestic students (26/29, 90%) studying at the undergraduate level (21/29, 72%) across a range of disciplines (eg, Health and Medicine and Arts and Social Sciences). Unlike most participants randomized to the intervention, engagement among this group was very high; participants accessed a mean of 10.86 (SD 2.37) modules, started a mean of 8.28 (SD 4.33) videos, and accessed a mean of 4.14 (SD 3.62) exercises.

#### Qualitative Categories

One category was identified from the interviews and open-ended survey responses regarding patterns of skill enactment among participants: (1) limited initial and ongoing skill enactment. Three categories were identified regarding the factors that influenced participants’ use of the program techniques: (1) relevance and perceived fit, (2) practicality and ease of implementation, and (3) exploring novel approaches and reinforcing familiar ones.

#### Limited Initial and Ongoing Skill Enactment

Overall, participants’ initial engagement with the exercises designed to facilitate the practice of the therapeutic techniques was limited. Where participants did engage, most stated that they tried only some of the exercises, and several participants described a pattern of engagement that lacked depth in terms of the time, effort, or attention they gave to the exercises. For some, this involved reading through the exercises without any or limited further engagement:

Like the exercises and things like that, like I read through them. I didn’t really, I didn’t do any of the exercises myself, but I did...I did read through them and look at them and they seemed pretty, pretty good.

M1, interview

Others described completing the exercises, but not in a “fully focused” way [M3, interview], and some commented that their engagement did not go beyond their initial interaction with the exercise in the web-based platform, especially if the exercise included a writing component:

For ones that required actually writing things down, if I had something in the moment that I wanted to...that I thought of as I was, like, reading through the exercises then I put them down. But I didn’t, like you know, take it as homework and adopt it within my lifestyle.

F2, interview, writing exercises

In general, participants did not describe continuing to enact skills from the program after initially interacting with the web-based exercises, and one student noted that they were not “consistent” in their use of skills (F5, interview). At the same time, participants described finding the techniques they did try, which included a mixture of thought records, pleasant events schedules, behavioral experiments for social anxiety, procrastination strategies, body functionality appreciation, or mindfulness exercises, to be practical and useful, and several stated that they would use them in the future if needed:

But I can probably look back at that. So, I’ve downloaded all of them though, for like later reference if I need them.

F6, interview, behavioral experiments for social anxiety

#### Relevance and Perceived Fit

Several participants described the relevance of certain strategies or techniques to their specific symptoms or challenges as a key influence on their decisions to initially engage with the exercises. Techniques that were not seen to be relevant to students’ symptoms were less likely to be used, whereas those that were more relevant were more likely to be used:

I think at the time, it probably applied to me a bit more. I think that I was probably feeling a bit low in the social skills area of life. Yeah.

F8, interview, behavioral experiment for social anxiety

Perceived compatibility between an individual’s personal characteristics, their goals, and the techniques taught in the intervention was also mentioned by some participants as positively or negatively influencing their use of specific techniques. For example, one participant commented on the fit between a cognitive exercise (a thought record) and their personal characteristics:

And it...it kind of...I am a very logical and, like, a person who likes to process things. So, like, I liked the layout of it, that you could fill in a table and see...work it out.

F1, interview, thought record exercise

Although mentioned less often, another participant stated that their use of the techniques was motivated by their preexisting mental health goals:

Okay, so pretty much like I’d already been wanting to meditate. And I feel like the videos just like reinforced it.

M4, interview, mindfulness exercise

To address issues of relevance and fit, participants recommended tailoring or personalizing program content, for example, by modifying content according to the needs of the student or by highlighting that users can work through the exercises based on their needs and preferences. One participant suggested that “the exercises might be improved by having a few variations by which one might manage their issues” within each module [M5, free-text response].

#### Practicality and Ease of Implementation

The degree to which participants perceived the strategies to be practical and easy to implement in everyday life also affected their initial willingness to engage with and enact techniques. For example, techniques that were seen as enjoyable, rewarding, and having a tangible impact on mood (such as the pleasant events schedule) were easier to implement for some participants:

So then incorporating, I think it was like one achievable task that will give you a sense of achievement and one task for fun. And I’m not used to like scheduling in things just for fun. So that felt really rewarding.

F4, interview, pleasant events schedule

Conversely, some participants stated that they were less likely to use exercises that had a significant web-based writing component or that involved multiple steps (such as the thought records):

Yeah, there were...there was a lot to like write down and it was maybe if it was like a thought exercise like with just like a couple of steps to do I maybe would have done it more but just because I’m not at my laptop all day.

F3, interview, thought record exercise

Other factors that made it challenging to implement the exercises, in general, and in terms of particular topics, included forgetting and lack of time due to other demands:

Ah, time, I suppose...But I’ve had a very kind of busy schedule, which means I probably should have prioritized it, but I didn’t as much as I should have.

F6, interview, body functionality appreciation exercise

...I did download them, but I just forgot about them.

F13, free-text response

Participants indicated that changing the presentation of the thought records to be less cognitively demanding (such as by breaking down the exercise into smaller steps) and providing calls to action (eg, using weekly reminders) could facilitate engagement. Several participants also suggested that the exercises should be more interactive:

Possibly make them a bit more interactive—maybe add a challenge for the week or something (to help with motivation for these exercises/activities).

F19, free-text response

#### Exploring Novel Approaches and Reinforcing Familiar Ones

Participants also stated that their use of certain strategies or techniques was shaped by previous exposure. Several participants commented that they used strategies that were new to them because the concepts sparked curiosity and motivated them to try out new ways of coping or relating to themselves:

And I don’t know why I practiced that one in particular, because I’m happy with my body image. But it was just a really interesting notion of ‘don’t think about, like how your body looks, think about how it performs,’ and things like that.

F5, interview, body functionality appreciation exercise

In contrast, some participants indicated that they used strategies with which they were already familiar because this reinforced previous knowledge and behaviors:

There was like, there was one of them...that is like, you know, I think I’ve heard it before. I think it’s like a common thing. But it was just good to reinforce it. I guess.

M2, interview, behavioral experiments for social anxiety

Improving sleep...yeah, yeah so, I already kind of try and do some of that but it’s helpful to kind of have a refresher.

F6, interview, sleep hygiene exercise

## Discussion

### Principal Findings

This mixed methods study aimed to examine skill enactment among university students participating in an RCT of a low-intensity video-based intervention (UVC-Lite). The intervention was not found to be effective in improving self-reported skill enactment. There was also no evidence to indicate that participants who reported greater skill enactment experienced greater improvement in symptoms. This finding is not surprising given that skill enactment remained relatively stable over time and because we did not observe an overall effect of the intervention on primary mental health outcomes in the main trial [40]. These results raise the possibility that it may be difficult to facilitate changes in skill enactment via the unguided use of brief transdiagnostic modules in a sample of university students with mild to moderate levels of distress. Several previous studies have demonstrated that DMHIs can lead to improvements in skill enactment, with some evidence indicating that these improvements mediate improvements in symptoms of depression and anxiety [35,36,38,63]. These factors may provide users with more opportunities to consolidate, practice, and benefit from program skills through increased repetition and reinforcement.

Use of the materials was also low in this trial (ie, approximately half of the participants either did not start any of the videos or accessed any of the activities). These results are comparable to previous studies of DMHIs for students and young people [64-66], including a recent study by Bolinski et al [67], which found that although most students started at least 1 session of an automated transdiagnostic intervention, they completed an average of only 2 (25%) out of the 8 mandatory modules. The low rates of program use in this study suggest that a key explanation for our null results may be insufficient exposure to the therapeutic content. Our subgroup analyses lent some support to this explanation, as contrasts indicated that the program may be effective in improving skill enactment at postintervention and 6-month follow-up for participants who accessed at least some of the intervention materials. However, this result should be interpreted with caution given that the analyses were post hoc and represented small effects, the overall model was not significant, and an effect was not observed at 3-month follow-up. Furthermore, improvements in skill enactment among this group also did not translate to improvements in depression or anxiety, perhaps because the analysis was not sufficiently powered to detect small effects that have been observed in other studies [36].

It is also possible that relatively high baseline levels of self-reported skill enactment (49.1%-83.6% of participants indicated that they used each of the skills, at least sometimes; median 70.8%, IQR 62.4%-78.5%) and low baseline severity of depression and anxiety symptoms affected our ability to detect change. We did find partial support for this idea in our post hoc analysis comparing skill enactment among participants who reported reliable improvement against those who did not. Higher skill enactment was reported at postintervention and 3-month follow-up among those who demonstrated reliable improvement in symptoms. However, there was no evidence to suggest that the intervention led to greater improvements in skill enactment in this group. Further investigation is needed to examine the conditions under which this intervention may be effective and to explore the relationship between skill enactment and outcomes in a larger sample of students with a higher need or more severe symptoms (ie, those with greater potential to experience reliable or clinically significant change).

The results of our qualitative analysis help to further elucidate the processes underlying skill enactment. Participants who provided qualitative data constituted a unique subset of users, as most had high levels of program use. However, even among this group, skill enactment was limited and often did not extend beyond students’ initial interactions with the web-based exercises. Instead, participants tended to highlight that they planned to use the skills if they experienced a worsening of symptoms. Similar experiences were expressed by participants in 2 previous studies of adolescent and adult users of DMHIs, wherein the authors observed that some users adopt a passive and reactive approach to applying treatment principles [39,68]. Both in these previous studies and our study, this approach appeared to be underpinned by participants’ judgments concerning their current and potential future need for strategies to address various mental health concerns, suggesting that tailoring intervention content and activities based on an initial assessment of symptoms may improve skill enactment. However, other studies have reported that students are not more likely to choose content matched to their symptoms based on clinical assessment [69,70], and some research has linked choice to improved outcome [71], which suggests that tailoring approaches may need to strike a balance between student-identified concerns and clinical recommendations.

In addition, participants also highlighted that they were motivated to use skills that were easily integrated into daily routines rather than those that involved a significant writing component (eg, thought record exercises). These findings are consistent with past research on the co-design and evaluation of DMHIs among young people, which found that this group has a preference for practical and easy to use information and an aversion to text-heavy content [72-74]. This might suggest that behavioral activities (eg, pleasant events scheduling) may be more useful among students with mild to moderate distress than cognition-focused exercises, consistent with the suggestions offered by Titov et al [32] that different actions may be important at different levels of mental health. Yet individual differences also appeared to have a substantial influence on participants’ decisions to enact certain skills, for example, some participants highlighted that their engagement decisions were underpinned by judgments regarding whether the approach was consistent with their personal characteristics (eg, approach to problem-solving and preexisting goals), and others pointed to the newness or, conversely, the familiarity of the strategy as positively influencing skill enactment.

Suggestions offered by participants to improve engagement with the therapeutic activities included changing the presentation of writing-based activities to be less cognitively demanding (eg, using tunneling features), providing reminders, and enhancing interactivity (eg, using weekly challenges). These results align broadly with the recommendations for persuasive system design provided by Baumel et al [27], who suggested that calls to action (eg, reminding and inspiring users to use a technique) and load reduction (eg, gradual presentation of information to reduce cognitive load) may help encourage users of digital interventions to integrate therapeutic activities in everyday life. Gamification elements, such as the use of points and reward systems or leaderboards, might also be used to enhance interactivity and support motivation among students who face barriers to skill enactment, with a previous RCT of an internet-based intervention finding that adult participants who received concurrent access to a smartphone app encouraging the completion of small challenges in daily life had greater skill enactment than those who did not have concurrent access to the app [75].

### Implications

This study was motivated by calls for research to measure and report indicators of meaningful and active engagement such as skill enactment in digital interventions [23,24,27]. However, low rates of program use made it challenging to draw meaningful conclusions regarding whether low-intensity interventions such as the UVC-Lite have the capacity to improve skill enactment among students, or about the potential effects of skill enactment on outcomes. Our results related to low program use and high dropout are common in several trials of digital CBT interventions for young people with relatively mild symptoms [18,76]. They suggest that a low perceived need for intervention among students with mild to moderate symptoms may undermine their motivation to fully engage with CBT-based programs. Lack of perceived need can reduce program uptake and participation [77], but it may also result in a lack of active engagement with therapeutic skills and limited ongoing behavior changes among those who do participate. This suggests that an important priority for future research would be to establish whether low-intensity programs can effectively improve levels of skill enactment and mental health outcomes among students with more severe symptoms. In addition, the potential for the large-scale implementation of unguided interventions in university settings suggests that another direction for future research would be to ascertain the most effective approaches for prevention and early intervention among students. Alternative approaches such as those that focus on preparation for difficult times or establishing healthy routines may be needed with this population. This might include not only mental health education to promote appropriate support seeking, but also well-being interventions (eg, mindfulness-based and positive psychological interventions), stress management programs, strength-based planning, lifestyle interventions, and CBT-based programs targeting less stigmatized challenges such as sleep disturbance.

The study also points to potential pathways for improving intervention design to promote skill enactment. Our qualitative findings related to individual differences in skill enactment suggest that 1 promising approach may be to tailor therapeutic content to the needs and preferences of individual users. For instance, real-time analysis of user behaviors could be used to dynamically tailor therapeutic recommendations and feedback [27], continuous engagement with certain activities might trigger recommendations for more complex activities, while a lack of engagement could trigger recommendations for alternative activities or feedback to address barriers. Tailoring therapeutic recommendations based on other user characteristics is also likely to be beneficial (eg, through assessment of user-identified concerns, goals, or existing mental health knowledge). However, this will require a better understanding of the processes underlying skill enactment among users of DMHIs, including factors that influence initial and ongoing cognitive and behavioral change in everyday life, as well as factors that affect the enactment of different types of skills, especially among different student groups. Qualitative methods involving consultation with end users and the application of structured frameworks of behavior change during data collection and interpretation (eg, theoretical domains framework [78]) may help address these aims.

### Strengths and Limitations

Strengths of the study include the large sample relative to other trials testing web-based interventions in tertiary student populations [79-81], recruitment of participants from universities across Australia rather than a single institution, and a focus on maximizing ecological validity. However, high ecological validity was also a limitation due to high rates of attrition and low adherence. This may have led to inaccurate estimates of the treatment effect, although we used rigorous MMRM models to account for all available data. Our use of qualitative data to supplement the quantitative analysis represents an additional strength. However, we relied on a self-selected sample of students to provide these data, nearly all of whom were highly engaged. This may have resulted in a sample skewed toward those who had predominantly positive experiences of the intervention, and our findings may not apply to students who did not use or only minimally used the intervention. Questions regarding skill enactment were also asked as part of a larger qualitative study designed to examine students’ views on implementing the program within universities, and thus we may not have captured other experiences related to skill enactment. Furthermore, we did not ask participants about potential factors other than the program that may have influenced skill enactment during the measurement period.

In addition, participation among males, gender-diverse students, and international students was low, which may limit the generalizability of the results to other populations. Although this reflects differences in the help-seeking and participation in mental health research more generally [82-84], ongoing efforts to improve participation are important to ensure DMHIs are acceptable and effective for these groups. Given the high number of international students attending university in Australia [85], future studies might also consider the utility of offering interventions in multiple languages to maximize accessibility for international and culturally and linguistically diverse students. We also relied on brief self-report scales to measure outcomes. Although assessments included validated measures of depression and anxiety, a bespoke scale was used to assess skill enactment as none of the standardized measures available at the time of trial design adequately captured the range of techniques covered in the UVC-Lite intervention [46,47,86]. Titov et al [32] have since developed and evaluated a comprehensive self-report questionnaire of mentally healthy actions that is not tied to a specific therapeutic approach and which may assist other researchers to evaluate skill enactment using a standardized approach. However, it will also be important for future studies to assess the fidelity of skill enactment (ie, the extent to which individuals enact skills in line with how they are taught), for example, through evaluations of homework or therapist ratings [30]. Moreover, delivery of the 3-month follow-up assessments was delayed by approximately 1 month for a proportion of participants due to an administrative error.

### Conclusions

This study indicated that a transdiagnostic low-intensity video-based program was not effective in improving skill enactment among university students with mild to moderate levels of psychological distress. Our null results may be due to several factors, including characteristics of the intervention, low program use, low baseline severity of participants’ symptoms, and high levels of baseline skill enactment. The results of our qualitative analysis suggested that initial and ongoing skill enactment was limited even among participants with high program use, and individual differences appeared to have a substantial influence on decisions to enact skills. Ongoing research with university students is needed to determine who can benefit from low-intensity digital interventions, what types of digital interventions may be most effective, and how we can improve engagement, both in terms of program use and skill enactment, to ensure that the potential benefits of DMHIs are realized. Qualitative and theory-informed research may lead to a better understanding of the mechanisms underlying skill enactment.

## Appendix

Multimedia Appendix 1 CONSORT-eHEALTH checklist (V 1.6.1).

Multimedia Appendix 2 Results of the exploratory factor analysis on skill enactment items.

Multimedia Appendix 3 Interview questions.

## Abbreviations

CBT: cognitive behavioral therapy
CONSORT-eHEALTH: Consolidated Standards of Reporting Trials of Electronic and Mobile Health Applications and Online Telehealth
DMHI: digital mental health intervention
GAD-7: Generalized Anxiety Disorder-7
MMRM: mixed model repeated measures
PHQ-9: Patient Health Questionnaire-9
RCT: randomized controlled trial
UVC-Lite: Uni Virtual Clinic Lite
