# Supplementary material for: Skill Enactment Among University Students Using a Brief Video-Based Mental Health Intervention: Mixed Methods Study Within a Randomized Controlled Trial
Source: JMIR Ment Health. 2024 Aug 21;11:e53794. doi: 10.2196/53794 (PMC11375386; doi:10.2196/53794)
Supplement: Multimedia Appendix 1 [file mental_v11i1e53794_app1.pdf]

CONSORT-EHEALTH Checklist V1.6.2 Report	Manuscript Number	53794

(based on CONSORT-EHEALTH V1.6), available at [http://tinyurl.com/consort-ehealth-v1-6].

Date completed

10/30/2023 1:21:33

by

Hayley Jackson

Skill Enactment Among University Students Using a Brief Video-Based Mental Health Intervention: Mixed Methods Study Within a Randomised Controlled Trial

TITLE

1a-i) Identify the mode of delivery in the title

Yes, the mode of delivery is identified in the title, “a Brief Video-Based Mental Health Intervention”.

1a-ii) Non-web-based components or important co-interventions in title

This item is not applicable to our study as there were no non-web-based or other co-interventions.

1a-iii) Primary condition or target group in the title

Yes, the target conditions are highlighted (i.e., “Mental Health”), as is the target group, (i.e., “university students”).

ABSTRACT

1b-i) Key features/functionalities/components of the intervention and comparator in the METHODS section of the ABSTRACT

Yes, “This study aimed to assess the effects of a low-intensity video-based intervention, Uni Virtual Clinic Lite (UVC-Lite), in improving skill enactment relative to an attention-control program (primary aim)…”

1b-ii) Level of human involvement in the METHODS section of the ABSTRACT

Yes, it was "self-guided", this is identified in the abstract.

1b-iii) Open vs. closed, web-based (self-assessment) vs. face-to-face assessments in the METHODS section of the ABSTRACT

Yes, they were recruited from “universities across Australia,” assessments were online.

1b-iv) RESULTS section in abstract must contain use data

Yes, “…UVC-Lite (n=243) or an attention-control program (n=244).” Non-significant change in use of therapeutic skills also reported, “($F_{3,215.36}$=0.50, P=.68)”. Program usage data is not reported in abstract as this is explored in-depth in the main results paper (see Louise M Farrer et al. A transdiagnostic video-based internet intervention to improve the mental health of university students: Randomised controlled trial of the Uni Virtual Clinic-Lite. J Med Internet Res (submitted). doi:10.2196/53598).

1b-v) CONCLUSIONS/DISCUSSION in abstract for negative trials

Yes, “The intervention did not improve skill enactment or mental health among students with mild to moderate psychological distress. Low adherence impacted our ability to draw robust conclusions regarding the intervention’s impact on outcomes.”

INTRODUCTION

2a-i) Problem and the type of system/solution

Yes, “several systematic reviews have indicated that DMHIs targeting tertiary students can be effective for symptoms of depression and anxiety when delivered under trial conditions … However, a recent systematic review of digital CBT programs targeting depression and anxiety identified a gap in the literature on skill enactment among young people [31], and none of the included studies explored change in skill enactment or its association with mental health outcomes among a university student sample.”

2a-ii) Scientific background, rationale: What is known about the (type of) system

Yes, “…the cognitive and behavioural changes a user makes in their everyday life as a result of intervention [27-31]…may be critical to understanding the impact of an intervention on outcomes. This is because most therapeutic programs are built on a theoretical understanding that positive change occurs by facilitating the uptake of adaptive cognitive and behavioural skills [27].”

Does your paper address CONSORT subitem 2b?

Yes, “we hypothesised that skill enactment would be higher in the UVC-Lite intervention condition than the attention control condition at post-intervention, 3- and 6-month follow-up assessments. We also expected that efficacy…would be greater for those in the intervention group with higher skill enactment, relative to the control group.”

METHODS

3a) CONSORT: Description of trial design (such as parallel, factorial) including allocation ratio

Yes, “Data for this study were collected as part of an RCT that examined the effectiveness of UVC-Lite in reducing depression and anxiety symptoms among university students with mild-moderate levels of psychological distress, relative to an attention-control condition.”

3b) CONSORT: Important changes to methods after trial commencement (such as eligibility criteria), with reasons

There were no major changes to the trial design following trial commencement.

3b-i) Bug fixes, Downtimes, Content Changes

No changes were made on the intervention or comparator during the trial. An administrative error that occurred during the trial is discussed in the Discussion section, “…delivery of the 3-month follow-up assessments was delayed by approximately 1 month for a proportion of participants due to an administrative error,” as well as in the main results paper.

4a) CONSORT: Eligibility criteria for participants

Yes, these were: “(1) enrolled at an Australian university and resided in Australia, (2) aged between 18 and 25 years, and (3) scored between 8 and 17 on the Distress Questionnaire–5 (DQ5)>”

4a-i) Computer / Internet literacy

We did not measure internet literacy, as it is assumed given the target population (university students).

4a-ii) Open vs. closed, web-based vs. face-to-face assessments:

Yes, “Trial participants were 487 undergraduate and postgraduate students, recruited and randomised into the trial between August 2021 and May 2022.” More details on recruitment are available in the main trial paper (Farrer et al., submitted)

4a-iii) Information giving during recruitment

Yes, “All participants provided informed consent by ticking a checkbox online after viewing an information sheet containing key details about the study.” Complete information on recruitment methods is available in the main trial paper (Farrer et al., submitted).

4b) CONSORT: Settings and locations where the data were collected

Yes, participants were “undergraduate and postgraduate students…enrolled at an Australian university…recruited online.”

4b-i) Report if outcomes were (self-)assessed through online questionnaires

Yes, “Quantitative data on skill enactment, depression symptoms, and generalised anxiety symptoms were obtained using online self-report questionnaires…”

4b-ii) Report how institutional affiliations are displayed

The trial was clearly identified as being conducted by the Australian National University.

5) CONSORT: Describe the interventions for each group with sufficient details to allow replication, including how and when they were actually administered

5-i) Mention names, credential, affiliations of the developers, sponsors, and owners

PJB, ALC, and LMF are co-developers of the UVC-Lite intervention. LMF was involved in the development of the original Uni Virtual Clinic intervention.

5-ii) Describe the history/development process

Yes, “Content for the intervention was drawn from The Uni Virtual Clinic, an online mental health intervention for university students that was shown to be effective in reducing symptoms of social anxiety and improving academic self-efficacy compared to a wait-list control group in a previous trial [40, 43].”

5-iii) Revisions and updating

Not applicable, this is the only version of UVC-Lite.

5-iv) Quality assurance methods

This item is not applicable.

5-v) Ensure replicability by publishing the source code, and/or providing screenshots/screen-capture video, and/or providing flowcharts of the algorithms used

Not relevant to the current study.		
**5-vi) Digital preservation**		
The intervention is not currently publicly available. It will be available to Australian university students in early 2024.		
**5-vii) Access**		
The eligibility criteria for the study are clearly identified in the Methods section. Also, “No incentives were offered for participation in the interview.”		
**5-viii) Mode of delivery, features/functionalities/components of the intervention and comparator, and the theoretical framework**		
Yes, the features and components of the intervention and comparator are described under “Intervention Condition: UVC-Lite” and “Attention Control Condition: General Health Information”.		
**5-ix) Describe use parameters**		
Yes, post-intervention assessments were “emailed to participants 6 weeks after completion of the baseline survey.” More information on use parameters is provided in the main trial paper (Farrer et al., submitted).		
**5-x) Clarify the level of human involvement**		
Yes, “Quantitative data…were obtained using online self-report questionnaires at baseline, post-intervention assessment (emailed to participants 6 weeks after completion of the baseline survey), and three- and six-month follow-ups (emailed to participants three and six months after receiving the post-intervention survey). Qualitative data were collected…through interviews conducted with participants following completion of post-intervention assessments.”		
**5-xi) Report any prompts/reminders used**		
Yes, “Participants received two email reminders to finish the post-intervention and follow-up surveys.”		
**5-xii) Describe any co-interventions (incl. training/support)**		
This item is not applicable, as no co-interventions were offered.		
**6a) CONSORT: Completely defined pre-specified primary and secondary outcome measures, including how and when they were assessed**		
Yes, full information on when outcomes were assessed is provided in the “Data Collection” section of the manuscript. The primary outcome (skill enactment) was assessed using “a list of 14 items developed to assess the frequency of enacting therapeutic techniques from the UVC-Lite program… Primary mental health outcomes were symptoms of depression, measured by the Patient Heath Questionnaire-9 (PHQ-9) [50], and generalized anxiety disorder, measured by the Generalized Anxiety Disorder Scale-7 (GAD-7) [51].”		
**6a-i) Online questionnaires: describe if they were validated for online use and apply CHERRIES items to describe how the questionnaires were designed/deployed**		
The PHQ-9 and GAD-7 have been previously used and validated in online studies. The bespoke skill enactment scale developed for this study was examined using exploratory factor analysis, see Multimedia Appendix 1.		
**6a-ii) Describe whether and how “use” (including intensity of use/dosage) was defined/measured/monitored**		
Yes, “JavaScript code was used to track intervention group participants’ uptake and usage behaviours…including number of modules accessed (i.e., whether participants clicked to open each module), number of videos started (i.e., >0% of the video watched), and number of therapeutic activities accessed (based on whether participants clicked through to access the exercise).” Complete information on program usage are reported in the main trial paper (Farrer et al., Submitted).		
**6a-iii) Describe whether, how, and when qualitative feedback from participants was obtained**		
Yes, this is detailed in the “Data Collection - Qualitative Data” sections.		
**6b) CONSORT: Any changes to trial outcomes after the trial commenced, with reasons**		
Yes, participants were “undergraduate and postgraduate students…enrolled at an Australian university…recruited online.”		
**7a) CONSORT: How sample size was determined**		
**7a-i) Describe whether and how expected attrition was taken into account when calculating the sample size**		
Full information on the power analysis used to determine sample size and recruitment targets is given in the main trial paper (Farrer et al., submitted).		
**7b) CONSORT: When applicable, explanation of any interim analyses and stopping guidelines**		
Yes, full information on when outcomes were assessed is provided in the “Data Collection” section of the manuscript. The primary outcome (skill enactment) was assessed using “a list of 14 items developed to assess the frequency of enacting therapeutic techniques from the UVC-Lite program… Primary mental health outcomes were symptoms of depression, measured by the Patient Heath Questionnaire-9 (PHQ-9) [50], and generalized anxiety disorder, measured by the Generalized Anxiety Disorder Scale-7 (GAD-7) [51].”		
**8a) CONSORT: Method used to generate the random allocation sequence**		
“A full description of the trial methods and primary outcomes, including information on allocation concealment, randomisation procedures, and blinding, is provided in the main trial paper (LM Farrer, PhD, unpublished data, October 2023 [41]).”		
**8b) CONSORT: Type of randomisation; details of any restriction (such as blocking and block size)**		
Simple randomisation was used. This information is provided in full in the main results paper (Farrer et al., submitted).		
**9) CONSORT: Mechanism used to implement the random allocation sequence (such as sequentially numbered containers), describing any steps taken to conceal the sequence until interventions were assigned**		
Participants were manually allocated to the trial conditions by an independent researcher according to a randomly generated sequence of integers to ensure allocation concealment. This information is provided in full in the main results paper (Farrer et al., submitted).		
**10) CONSORT: Who generated the random allocation sequence, who enrolled participants, and who assigned participants to interventions**		
An independent researcher not involved in the trial generated a random sequence of integers between 1 and 2 using https://www.random.org/. This information is provided in full in the main results paper (Farrer et al., submitted).		
**11a) CONSORT: Blinding - If done, who was blinded after assignment to interventions (for example, participants, care providers, those assessing outcomes) and how**		
**11a-i) Specify who was blinded, and who wasn’t**		
Trial researchers were blinded to treatment allocation, participants were blinded to whether they received the active or attention control intervention, and assessments were blinded. This information is provided in full in the main trial paper (Farrer et al., submitted).		
**11a-ii) Discuss e.g., whether participants knew which intervention was the “intervention of interest” and which one was the “comparator”**		
Participants were informed that they would be randomised to receive one of two programs: information and strategies for improving mental health (UVC-Lite) or information and strategies for improving general health (attention control). They were not provided with information about which program was expected to be more effective. This information is addressed in full in the main outcomes paper (Farrer et al., submitted).		
**11b) CONSORT: If relevant, description of the similarity of interventions**		
Yes, “Participants assigned to the attention control condition received bi-weekly e-mails containing a web link to a PDF document providing information on topics related to general health rather than mental health, approximately matched for completion time with the active intervention.”		
**12a) CONSORT: Statistical methods used to compare groups for primary and secondary outcomes**		
Yes, “Mixed model repeated measures (MMRM) analysis of variance (ANOVA) was conducted to compare mean scores on skill enactment between the intervention and control groups, with measurement occasion as a within-groups factor and condition as a between-groups factor… Evidence for moderation of primary outcomes (PHQ-9, GAD-7) by change in skill enactment was tested using MMRM ANOVAs with three-way interaction terms of time × condition × skill enactment.”		
**12a-i) Imputation techniques to deal with attrition / missing values**		
Yes, “MMRM techniques incorporate all available data under the assumption that data are missing at random [56].”		
**12b) CONSORT: Methods for additional analyses, such as subgroup analyses and adjusted analyses**		
Yes, “Sub-group analyses of intervention group participants who started one or more videos versus all of the control group were conducted to estimate the effectiveness of UVC-Lite among participants who accessed at least some of the therapeutic content.”		
RESULTS		
**13a) CONSORT: For each group, the numbers of participants who were randomly assigned, received intended treatment, and were analysed for the primary outcome**		
Yes, “…523 (18.3%) participants were initially screened into the trial, completed baseline measures, and were randomised to the UVC-Lite intervention (n=261) or attention control group (n=262). A further 36/523 (6.9%) participants were excluded after randomisation due to being older than the eligible age range...the majority (129/243, 53.1%; mean 2.48, SD 3.89) did not start any of the videos…” Full information is provided in the main results paper (Farrer et al., 2023)		
**13b) CONSORT: For each group, losses and exclusions after randomisation, together with reasons**		
Yes, see “Methods – Participants – Randomised Controlled Trial” as well as “Results -Sample Characteristics and Participation Rates”.		
**13b-i) Attrition diagram**		

Full information regarding attrition is provided in the main results paper (see the flow diagram and intervention adherence and usage data) (Farrer et al., submitted). Partial information is provided in this paper under “Program Usage”.		
**14a) CONSORT: Dates defining the periods of recruitment and follow-up**		
Yes, participants were“…recruited and randomised into the trial between August 2021 and May 2022. Follow-up data were collected by January 2023… interviews conducted with participants following completion of post-intervention assessments between April and August 2022.”		
**14a-i) Indicate if critical “secular events” fell into the study period**		
There were no known issues related to this.		
**14b) CONSORT: Why the trial ended or was stopped (early)**		
This item is not relevant as the trial was not stopped early.		
**15) CONSORT: A table showing baseline demographic and clinical characteristics for each group**		
Yes, please see Table 1 for baseline characteristics by condition.		
**15-i) Report demographics associated with digital divide issues**		
Yes, please see Table 1 for demographic information related to digital divide issues.		
**16a) CONSORT: For each group, number of participants (denominator) included in each analysis and whether the analysis was by original assigned groups**		
**16-i) Report multiple “denominators” and provide definitions**		
Yes, all participant numbers are listed and analyses were intention to treat using all available data.		
**16-ii) Primary analysis should be intent-to-treat**		
Yes, analyses were intention to treat using mixed models.		
**17a) CONSORT: For each primary and secondary outcome, results for each group, and the estimated effect size and its precision (such as 95% confidence interval)**		
Effect sizes are not reported as they are not likely to be meaningful.		
**17a-i) Presentation of process outcomes such as metrics of use and intensity of use**		
Operational definitions of our metrics of use are provided in the methods. See “Program Usage” for a detailed description of intervention use.		
**17b) CONSORT: For binary outcomes, presentation of both absolute and relative effect sizes is recommended**		
The trial was adequately powered and given we did not find significant effects, effect sizes were not calculated.		
**18) CONSORT: Results of any other analyses performed, including subgroup analyses and adjusted analyses, distinguishing pre-specified from exploratory**		
Sub-group analyses are clearly identified. Exploratory analyses are distinguished from pre-specified analyses.		
**18-i) Subgroup analysis of comparing only users**		
Subgroup analyses among participants who accessed one or more videos are clearly identified as exploratory.		
**19) CONSORT: All important harms or unintended effects in each group**		
Full information on possible harms or unintended effects are reported in the main results paper (Farrer et al., submitted).		
**19-i) Include privacy breaches, technical problems**		
Yes, “…delivery of the 3-month follow-up assessments was delayed by approximately 1 month for a proportion of participants due to an administrative error.” Complete information on this administrative error is describe in the main trial paper (Farrer et al., submitted).		
**19-ii) Include qualitative feedback from participants or observations from staff/researchers**		
Yes, this study reports partial dataset analysis (qualitative) of participants experiences and motivations for trying the therapeutic techniques. An additional qualitative study that reports on other qualitative data collected during the interviews and surveys is also currently in preparation.		
DISCUSSION		
**20) CONSORT: Trial limitations, addressing sources of potential bias, imprecision, multiplicity of analyses**		
**20-i) Typical limitations in ehealth trials**		
Yes, see limitations section of the manuscript. The discussed limitations include limited participation by males, gender-diverse and international students, the use of self-report measures, administrative errors, and attrition.		
**21) CONSORT: Generalisability (external validity, applicability) of the trial findings**		
**21-i) Generalizability to other populations**		
Yes, this was a real-world trial conducted in universities across Australia, however, “…participation among males, gender-diverse students, and international students was low, which may limit the generalisability of the results to other populations		
**21-ii) Discuss if there were elements in the RCT that would be different in a routine application setting**		
As the study was designed to be a real-world implementation RCT, very little would be different other than developing an official website to host the videos.		
**22) CONSORT: Interpretation consistent with results, balancing benefits and harms, and considering other relevant evidence**		
**22-i) Restate study questions and summarize the answers suggested by the data, starting with primary outcomes and process outcomes (use)**		
Yes, “This mixed methods study aimed to examine skill enactment among university students participating in an RCT of a low-intensity video-based intervention (UVC-Lite). The intervention was not found to be effective in improving self-reported skill enactment.”		
**22-ii) Highlight unanswered new questions, suggest future research**		
Yes, see “Implications”. For example: “…an important priority for future research would be to establish whether low-intensity DMHIs can effectively improve levels of skill enactment and mental health outcomes among students with more severe symptoms. In addition, the potential for the large-scale implementation of unguided interventions in university settings suggests that another direction for future research would be to ascertain the most effective approaches for prevention and early intervention.”		
Other information		
**23) CONSORT: Registration number and name of trial registry**		
Yes, “The trial was registered at the Australian New Zealand Clinical Trials Registry (ACTRN12621000375853).”		
**24) CONSORT: Where the full trial protocol can be accessed, if available**		
The trial protocol was not published.		
**25) CONSORT: Sources of funding and other support (such as supply of drugs), role of funders**		
“Funding for this project was provided by Australian Rotary Health. HMJ is supported by an Australian Government Research Training Program Scholarship and the Grace Groom Memorial Scholarship (2021). LMF is supported by an Australian Research Council Discovery Early Career Researcher Award (ARC DECRA) DE190101382. ALC is supported by a National Health and Medical Research Council (NHMRC) Fellowship (1173146). The funders were not involved in the writing, reviewing, or approval of the manuscript, nor were they involved in the decision to submit the manuscript for publication.”		
**X26-i) Comment on ethics committee approval**		
Yes, “The trial was registered at the Australian New Zealand Clinical Trials Registry (ACTRN12621000375853) and the ethical aspects of the research were approved by the Australian National University Human Research Ethics Committee (protocol #2020/412).”		
**x26-ii) Outline informed consent procedures**		
Yes, “All participants provided informed consent by ticking a checkbox online after viewing an information sheet containing key details about the study.” More information on procedures used to obtain informed consent are provided in the main results paper (Farrer et al., submitted).		
**X26-iii) Safety and security procedures**		
Participants who contacted the research team in distress were followed-up by LMF, a registered psychologist. In addition, “Students who were ineligible due to high levels of psychological distress were provided with help-seeking resources.” Full information on safety procedures used throughout the trial are given in the main results paper (Farrer et al., submitted).		
**X27-i) State the relation of the study team towards the system being evaluated**		
As listed, some of the authors were involved in the development of the original Uni Virtual Clinic and/or the UVC-Lite.		
