# Supplementary material for: Skill Enactment Among University Students Using a Brief Video-Based Mental Health Intervention: Mixed Methods Study Within a Randomized Controlled Trial
Source: JMIR Ment Health. 2024 Aug 21;11:e53794. doi: 10.2196/53794 (PMC11375386; doi:10.2196/53794)
Supplement: Multimedia Appendix 2 [file mental_v11i1e53794_app2.docx]

**Multimedia Appendix 2:** **Results of the exploratory factor analysis on skill enactment items.**

Prior to conducting the main analyses, the 14 skill enactment items were subjected to EFA with principal axis factoring. We removed a single item (“I noticed myself thinking unhelpful or unrealistic thoughts”) from the item pool due to a low and negative factor loading (-0.15). Sampling adequacy for the remaining 13 items was verified by a Kaiser-Meyer-Olkin value of .88, and all diagonal elements on the anti-image correlation matrix exceeded .5 (range .76–.91). Bartlett’s test of sphericity indicated adequate intercorrelations between items, *χ^2^*_78_=1535.9, *P*<.001. Three factors were initially extracted with eigenvalues greater than 1, explaining 38.7% of the variance. Inspection of the scree plot revealed a clear break after the first factor. Parallel analysis with 1000 datasets specified on a permutation of the raw dataset using O’Connor’s SPSS syntax [47] generated 2 eigenvalues: 4.41 and 1.33. Based on Cattell’s scree test [48] and the presence of a primary factor accounting for about 4.5 times as much variance as the second factor, one factor was retained accounting for 28.5% of the total variance. The construct was interpreted as representing overall skill enactment. All item loadings exceeded .32 (range: .42 to .63), indicating adequate loadings on the factor [49].

**Table 1.** Items, item-total correlations, extracted communalities, and factor loadings for the 13-item skill enactment scale (N=487).

| Item | Item order | Item-total correlation | Extracted communalities | Item loading |
| --- | --- | --- | --- | --- |
| I noticed and challenged my perfectionistic thoughts. | Item 6 | 0.57 | 0.39 | 0.63 |
| I noticed and appreciated what my body can do, rather than how it looks. | Item 13 | 0.56 | 0.39 | 0.62 |
| I thought about my reasons to live, what makes me strong, and what supports I could access if I needed to. | Item 14 | 0.53 | 0.34 | 0.59 |
| I focused on the present moment. | Item 7 | 0.52 | 0.33 | 0.57 |
| I reached out to others for support when I needed it. | Item 10 | 0.50 | 0.30 | 0.55 |
| I challenged my thinking to be more realistic and helpful. | Item 3 | 0.49 | 0.29 | 0.54 |
| I practiced healthy sleep habits (e.g., went to bed at the same time each night, removed devices from the bedroom). | Item 8 | 0.48 | 0.28 | 0.53 |
| I increased my opportunities for social interaction. | Item 11 | 0.48 | 0.27 | 0.52 |
| I practiced healthy social media habits (e.g., limited time spent scrolling, took a break from social media). | Item 12 | 0.47 | 0.27 | 0.52 |
| I used strategies to manage my time effectively. | Item 5 | 0.45 | 0.24 | 0.49 |
| I reduced anxious feelings by slowing my breathing or focusing on the things around me. | Item 4 | 0.43 | 0.23 | 0.48 |
| I took part in social situations, even if I felt uncomfortable. | Item 9 | 0.42 | 0.21 | 0.46 |
| I made time for activities that make me feel better. | Item 1 | 0.38 | 0.17 | 0.42 |
|  |  |  |  |  |
| Eigenvalue |  |  |  | 4.41 |

*Note:* One item (“I noticed myself thinking unhelpful and realistic thoughts”, Item 2) was removed from the scale due to a low and negative item-total correlation (-0.13) and factor loading (-0.15).
