# Supplementary material for: Skill Enactment Among University Students Using a Brief Video-Based Mental Health Intervention: Mixed Methods Study Within a Randomized Controlled Trial
Source: JMIR Ment Health. 2024 Aug 21;11:e53794. doi: 10.2196/53794 (PMC11375386; doi:10.2196/53794)
Supplement: Multimedia Appendix 3 [file mental_v11i1e53794_app3.docx]

Interview questions:

*Thank you for agreeing to participate in this interview. Before we start, I’ll just go through some housekeeping details.*

*You can stop the interview at any time, and you can refuse to answer any questions you like, just let me know. I’m audio-recording the interview (I’m not making a video recording), and I’ll delete the recording after the interview has been transcribed.*

*Also, it’s important to protect your privacy, so please try not to use names or indicate the name of your university. But if you do, don’t worry, I’ll delete these details from the transcript.*

*The questions I’m going to ask are about your thoughts on the video modules you completed in the trial, and their usefulness in a university environment. We’re particularly interested in your general thoughts or ideas about how best to provide these videos to students at university.*

First, I’d just like to quickly ask about your use of the videos:

1. How many of the videos did you watch? You don’t need to give an exact number, an estimate is fine (*participants don’t need to say exact number: broad indicators like ‘none’, ‘a few’, ‘some’, ‘most’, ‘all’ are fine*)
2. What did you think of the videos?
   1. Prompts: Did you find them helpful/informative? What did you think of their quality?
3. Did you practice any of the exercises that went along with the videos? Which ones and why? Was there a reason you didn’t try the other exercises?
4. How well did the videos meet your needs? e.g., reflecting on the reasons you wanted to take part in the trial … did the videos give you what you were looking for?
5. Did you experience any technical issues when trying to access the videos?

Next, I’d like to ask your thoughts about how best to provide these videos to university students:

1. Do you have any ideas about how these videos and exercises could be best delivered to university students?
   1. Prompt: How would you prefer to access these videos in a university setting? E.g., potential delivery modes: via official university student platforms, social media, email, text, residential halls
   2. Prompt: What are some ways that universities and staff could assist with delivering the videos?
   3. Prompt: Who in the university should be responsible for delivering or assisting students with using a program like this?
2. Are there any barriers to these videos being used by university students or being delivered in a university setting? How could these barriers be overcome?
3. Who do you think these videos could be particularly useful for?
   1. Do you think there might be any groups of students who might find it hard to access/engage with the videos? How could we help them to access/engage with the videos?
   2. Is there anything that might have helped you to engage more with the videos?

Are there any more ideas, thoughts or feedback that you would like to share?

Thank you!
